# Predictive value of systemic immune inflammation index combined with coagulation index in traumatic coagulopathy in patients with severe trauma

**DOI:** 10.5937/jomb0-51285

**Published:** 2025-01-24

**Authors:** Wang Chen, Huang Yongyong, Liao Shiyun, Song Jiming

**Affiliations:** 1 Longhua District Central Hospital of Shenzhen, Emergency Department, Shenzhene, China; 2 Longhua District Central Hospital of Shenzhen, Surgical Anesthesiology Department, Shenzhen, China

**Keywords:** systemic immune inflammatory index, coagulation index, severe trauma, traumatic coagulopathy, sistemski imunološki inflamatorni indeks, indeks koagulacije, teške traume, traumatska koagulopatija

## Abstract

**Background:**

Traumatic coagulopathy (TIC) poses a significant challenge in the management of severe trauma cases. Early identification of TIC and its risk factors is vital for initiating timely interventions. The systemic immune inflammation index (SII), a composite marker of inflammation and immune response, alongside conventional coagulation indices, may hold promise in predicting TIC. Here, this study aimed to evaluate the predictive value of combining SII with coagulation indices for TIC in severe trauma patients, with the goal of enhancing early detection and guiding prompt therapeutic strategies.

**Methods:**

The clinical data of patients with severe trauma treated in our hospital from January 2022 to December 2022 were retrospectively selected. According to the outcome of TIC, the patients were divided into TIC group (n = 50) and non-TIC group (n = 50). The general data, SII and individual indexes of the two groups were compared, and the influencing factors of TIC were analyzed by multivariate Logistics regression. ROC analysis of SII combined with blood coagulation index to predict traumatic coagulation in patients with severe trauma.

**Results:**

There was no significant difference in general data between the two groups. SII in TIC group was significantly higher than that in non-TIC group. neutrophil count (NEU), platelet count (PLT), lymphocyte count (LYM), activated partial thromboplastin time (APTT), prothrombin time (PT), fibrinogen (FIB) level, and D-Dimer (D-D) level in TIC group were higher than those in non-TIC group, while LYM, FIB was lower than that in non-TIC group. The logistic regression analysis showed that APTT, D-Dimer, FIB, PT, and SII were independent factors that significantly influenced TIC. The area under the curve of TIC in patients with severe trauma with SII combined with coagulation index was 0.883, and the standard error was 0.032 (95%CI:0.8195~0.9461). The best cut-off value was 0.65. The sensitivity and specificity were 80.3, 84.2 respectively.

**Conclusions:**

SII combined with coagulation index has high predictive value for TIC in patients with severe trauma. By monitoring these indexes, we can more accurately predict the occurrence of TIC and take effective treatment measures in time.

## Abstract (sr)

**Uvod:**

Traumatska koagulopatija (TIC) predstavlja značajan izazov u lečenju teških traumatskih slučajeva. Rana identifikacija TIC-a i njegovih faktora rizika je od vitalnog značaja za započinjanje pravovremenih intervencija. Indeks sistemske imune inflamacije (SII), kompozitni marker upale i imunološkog odgovora, zajedno sa konvencionalnim indeksima koagulacije, može koristiti u predviđanju TIC-a. Ovde je ova studija imala za cilj da proceni prediktivnu vrednost kombinovanja SII sa indeksima koagulacije za TIC kod pacijenata sa teškim traumama, sa ciljem poboljšanja ranog otkrivanja i vođenja brzih terapijskih strategija.

**Metode:**

Retrospektivno su odabrani klinički podaci pacijenata sa teškom traumom lečenih u našoj bolnici od januara 2022. do decembra 2022. godine. Prema ishodu TIC-a, pacijenti su podeljeni u TIC grupu (n = 50) i ne-TIC grupu (n = 50). Upoređeni su opšti podaci, SII i pojedinačni indeksi dve grupe, a faktori uticaja na TIC analizirani su multivarijantnom logističkom regresijom. ROC analiza SII u kombinaciji sa indeksom koagulacije krvi za predviđanje traumatske koagulacije kod pacijenata sa teškom traumom.

**Rezultati:**

Nije bilo značajne razlike u opštim podacima između dve grupe. SII u TIC grupi je bio značajno veči nego u grupi bez TIC. Broj neutrofila (NEU), broj trombocita (PLT), broj limfocita (LIM), aktivirano parcijalno tromboplastinsko vreme (APTT), protrombinsko vreme (PT), nivo fibrinogena (FIB) i nivo D-dimera (D-D) u TIC grupi su bili veči od onih u ne-TIC grupi, dok je LIM, FIB bio niži od onih u ne-TIC grupi. Logistička regresiona analiza je pokazala da su APTT, D-dimer, FIB, PT i SII nezavisni faktori koji su značajno uticali na TIC. Površina ispod krive TIC kod pacijenata sa teškom traumom sa SII u kombinaciji sa indeksom koagulacije bila je 0,883, a standardna greška 0,032 (95%CI: 0,8195~0,9461). Najbolja granična vrednost bila je 0,65. Osetljivost i specifičnost su 80,3, odnosno 84,2.

**Zaključak:**

SII u kombinaciji sa indeksom koagulacije ima visoku prediktivnu vrednost za TIC kod pacijenata sa teškom traumom. Pračenjem ovih indeksa možemo preciznije predvideti pojavu TIC-a i na vreme preduzeti efikasne mere lečenja.

## Introduction

Traumatic coagulopathy (TIC) is a disorder affecting the coagulation process that occurs following severe trauma [1]. Research has revealed that among patients suffering from severe trauma, the incidence of TIC stands at roughly 25%–35%, with affected individuals exhibiting a heightened propensity for blood loss, increased necessity for blood transfusions, and a greater risk of encountering multiple organ failure when contrasted with trauma patients who do not present TIC [2]. Early recognition and understanding of the risk factors associated with TIC can lead to timely interventions that positively impact patient treatment and prognosis [3]. Therefore, investigating and analyzing predictive indicators related to TIC holds significant clinical value. Currently, the prediction of traumatic coagulation in patients with severe trauma primarily relies on the patients’ medical history, physiological parameters, and laboratory test results, but these methods lack accuracy and are prone to bias [4]. The systemic immune inflammatory index (SII), which is based on peripheral blood neutrophils, platelets, and lymphocytes, serves as a useful reference for clinical assessments of disease progression, prognosis, and treatment effectiveness [5]. SII has been demonstrated to possess clinical prognostic value in malignant tumors. Coagulation indexes, including activation of neutrophil count (NEU), platelet count (PLT), lymphocyte count (LYM), activated partial thromboplastin time (APTT), prothrombin time (PT), fibrinogen (FIB) level, and D-Dimer (D-D) level, have specific normal ranges and clinical implications, providing direct insights into the functional state of the coagulation system [6]. Abnormal changes in these coagulation indexes can indicate the presence of underlying diseases. However, limited studies have examined the application of SII and coagulation indexes in TIC among patients with severe trauma. This study aims to investigate the predictive value of combining SII with coagulation parameters in patients with severe trauma for TIC.

## Materials and methods

### Subjects

A retrospective selection was conducted on the clinical data of severe trauma patients admitted to our hospital between January 2022 and December 2022. Inclusion criteria consisted of the following: (1) Patients meeting the diagnostic criteria for severe trauma patients [7] with an ISS score of 16 points. (2) Patients with complete clinical data. (3) Patients without a medication history of drugs interfering with coagulation. Exclusion criteria included: (1) Patients with other serious organ diseases. (2) Patients with mental disorders. (3) Patients with hematological disorders. (4) Patients with a history of immunosuppressant use.

### Grouping method

Based on the Chinese Expert Consensus on the Diagnosis and Treatment of Traumatic Hyper coagulability [8], it was observed that the APTT or PT showed a 1/2 increase compared to the normal range. Additionally, FIB levels were less than 1 g/L, indicating the presence of TIC. A total of 100 patients were categorized into two groups based on the presence or absence of TIC: 50 patients in the TIC group and 50 patients in the non-TIC group.

### Data collection channel

A cohort of 100 individuals was gathered using the electronic medical record system, and their basic information was documented upon admission. Venous blood samples were obtained to examine the following parameters upon admission: NEU, PLT, LYM, APTT, PT, FIB level, and D-D level. The selection of specific coagulation parameters—APTT, PT, FIB, and D-Dimer—is grounded in their established roles in assessing different aspects of the coagulation cascade and their clinical significance in TIC, as previously reported [6]. The SII was calculated using the formula SII = neutrophil count × platelet count / lymphocyte count.

### Outcome measures

(1) The goal is to assess and contrast the overall data and SII of patients in two separate groups. (2) The objective is to evaluate and compare the specific indicators of patients in two distinct groups. (3) Using multi-factor logistic regression, this study aims to examine the influential factors of TIC. (4) By utilizing ROC analysis on the systemic immune inflammatory index in conjunction with the coagulation index, the intention is to predict the occurrence of traumatic coagulation disease in patients with severe trauma. Additionally, this analysis seeks to determine the area under the curve, sensitivity, and specificity.

### Statistical analysis

The collected data underwent analysis using Statistic Package for Social Science (SPSS) 27.0 (IBM, Armonk, NY, USA). The normally distributed data were represented as x̄±S. To compare the data, an independent sample t-test was employed. On the other hand, count data were presented as either the number of cases or rates. For comparison, the χ^2^ test or Fisher’s exact method was conducted. Single factor and binary Logistics regression analyses were conducted to examine the influencing factors of TIC in severe trauma patients. To assess the predictive value of the combined SII and coagulation index for TIC in severe trauma patients, an ROC curve was utilized. A significance level of P<0.05 was employed.

## Results

### General information and SII status of patients in both groups

There was no statistically significant difference in overall data among TIC and non-TIC groups (P>0.05). The SII in the TIC group exhibited a substantially greater value compared to the non-TIC group (P<0.001), as indicated in Table 1.

**Table 1:** Analysis of general data and SII between the two groups.

Index	cases	Gender male (female)	Years (age)	SII
TIC group	50	35 (15)	45.41±5.12	1325.45±341.26
Non-TIC group	50	37 (13)	46.26±5.36	1081.11±311.37
t/X^2^		0.198	0.811	3.740
P		0.906	0.419	0.000

### Comparison of individual indicators between the two groups

In the TIC group, the NEU, PLT count, APTT, Dmurd, and PT levels were significantly higher in the TIC group compared to the non-TIC group (P<0.05). Conversely, the LYM count and FIB levels were significantly lower in the TIC group compared to the non-TIC group (P<0.05), as indicated in Table 2.

**Table 2:** Comparison of individual indexes between the two groups.

Index	TIC group (n=50)	Non-TIC group (n=50)	t	P
NEU (×10^9^/L)	7.79±3.61	6.58±1.62	2.162	0.033
PLT (× 10^9^/L)	20.16±5.22	12.34±5.26	7.462	0.000
LYM (× 10^9^/L)	1.66±0.08	1.74±0.09	4.698	0.000
APTT (s)	63.76±19.46	54.43±18.26	2.472	0.015
D-D (ng/mL)	50.28±17.45	36.11±15.44	4.300	0.000
FIB (g/L)	1.76±0.04	2.05±0.11	17.520	0.000
PT (s)	24.14±10.13	19.16±10.41	2.424	0.017

### Multivariate Logistics regression analysis of influencing factors of TIC

In the analysis of patients with severe trauma, the independent variables, namely APTT, D-Dimer, FIB, PT, and SII, were assigned with their respective actual values. The dependent variable in this case was the TIC outcome, which was classified as TIC=1 for diagnosed cases and TIC=0 for non-TIC cases. Through logistics regression analysis, it was determined that APTT, D-Dimer, FIB, PT, and SII were independent factors that significantly influenced TIC in patients with severe trauma (P<0.05) (Table 3).

**Table 3:** Multivariate Logistics regression analysis of influencing factors of TIC.

Risk factors	β	SE	Ward	OR	95%CI	P
APTT	0.745	0.264	7.959	2.106	1.255~3.533	<0.001
D-Dimer	0.050	0.021	5.611	1.051	1.009~1.095	<0.001
FIB	0.713	0.277	6.634	2.041	1.186~3.513	0.011
PT	0.004	0.012	0.111	1.004	0.981~1.028	0.010
SII	0.021	0.011	3.570	1.021	0.999~1.043	<0.001

### Predictive value of ROC Analysis of SII combined with Coagulation Indexes in predicting TIC in patients with severe Trauma

**Figure 1:**
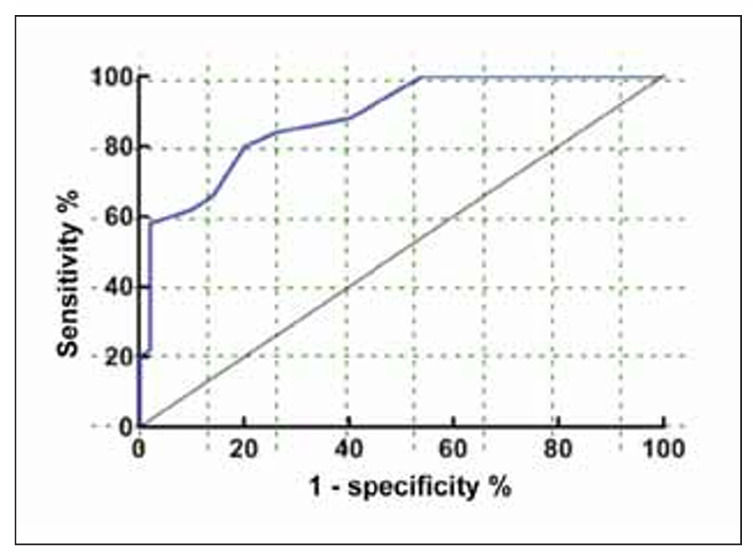
Predictive value of ROC Analysis of SII combined with Coagulation Indexes in predicting TIC in patients with severe Trauma.

The findings from the ROC analysis demonstrated that the combination of SII and coagulation index yielded an AUC for predicting TIC of 0.883, with a standard error of 0.032. The 95% confidence interval for the AUC ranged from 0.8195 to 0.9461. Moreover, the optimal cut-off value was identified as 0.65. The sensitivity and specificity of this model were 80.3% and 84.2% respectively. Figure 1

## Discussion

Increased bleeding, blood transfusion, multiple organ failure, and death are frequently observed in cases of TIC [9]. The mechanism underlying this condition is intricate, involving various factors. Following severe trauma, platelet activation and initiation of the coagulation cascade reaction can occur due to tissue injury and hemorrhagic shock [10]. Platelet activation results in the formation of an initial platelet clot, which serves to amplify the coagulation cascade, causing a thrombin burst and subsequent cleavage of fibrinogen into fibrin [11]. Fibrin can be degraded into soluble fibrin degradation products through the action of fibrinolytic enzymes. Although it is a common complication in patients with severe trauma, it can be partially prevented and treated [12] [13]. This study demonstrates that the combination of SII and blood coagulation indexes can serve as a predictive tool for the occurrence of TIC in patients with severe trauma, offering diagnostic value to some extent.

The findings indicated a significant increase in the SII of the TIC group compared to the non-TIC group. Additionally, the TIC group exhibited higher levels of NEU, PLT, APTT, Dmure D, and PT, while lower levels of LYM count and FIB. In their study, they also observed a strong correlation between APTT, D, PT, PLT, and FIB in predicting the prognosis of TIC patients. The analysis suggests that, in cases of TIC, neutrophils play a crucial role in immune response and inflammation. Neutrophils migrate to the injured area, aiming to eliminate infection and facilitate tissue repair [14]. Injured bodies trigger rapid activation and consumption of platelets, potentially leading to immune system suppression. As a result, NEU increases, while PLT and LYM count decrease. The Systemic Immune-Inflammation Index (SII), which reflects the inflammatory response and immune status of the body, is closely associated with NEU, PLT, and LYM count, thus providing a comprehensive assessment [15]. Severe trauma often places the body under high stress, triggering a robust activation of inflammatory and immune responses. This excessive activation can induce abnormal activation of the blood coagulation system and the subsequent development of coagulation disorders. Consequently, the body’s inflammatory and immune responses intensify, thereby increasing the risk of TIC and leading to an elevation in SII [16]. APTT and PT, commonly used to assess the function of the common coagulation pathway, serve as reliable indicators in evaluating coagulation function. In cases of TIC, the prolongation of APTT and PT occurs as a result of vascular endothelial damage and platelet activation caused by tissue injury and inflammation, which in turn triggers the initiation of the coagulation cascade [17] [18]. D-dimer, a product formed when plasmin initiates the degradation of fibrin into soluble fibrin, serves as an indicator of the activation of the common coagulation pathway and the breakdown of fibrin under the influence of fibrinolytic enzymes [19]. Hence, the elevation of D-dimer levels is observed in TIC. Fibrinogen, a crucial protein in the common coagulation pathway, is often depleted due to inflammatory reactions and liver function impairment. In cases of severe trauma, the concentration of FIB decreases as a result of the stress response, inflammatory reactions, and tissue injury [20].

Logistic regression analysis was employed to investigate the impact of these parameters on TIC in order to delve deeper into their influence. The findings indicated that APTT, D-Dimer, FIB, PT, and SII autonomously influenced TIC in patients with severe trauma. This implies that these markers possess significant predictive value and can serve as independent prognosticators. To further illustrate the predictive value of these markers, ROC analysis was conducted on SII in conjunction with blood coagulation indexes. The results unveiled an AUC of 0.883 and a standard error of 0.032 (95%CI: 0.8195~0.9461) for the combined use of SII and coagulation indexes in severe trauma patients. Additionally, with a best cutoff value of 0.65, the sensitivity and specificity were determined to be 80.3% and 84.2%, respectively, indicating the model’s high accuracy in forecasting the occurrence of TIC. The amalgamation of SII and blood coagulation indexes enables a more comprehensive assessment of the coagulation status in severe trauma patients [21]. SII signifies the systemic immune inflammatory response, whereas blood coagulation indicators reflect the functionality of the coagulation system. The amalgamation of these two aspects leads to a more accurate prediction of TIC and offers clinicians more valuable reference information [22].

The current study exclusively examined the predictive efficacy of SII in combination with blood coagulation index for TIC in patients with severe trauma. Nonetheless, the small sample size involved in this study may have introduced certain bias. Consequently, the reliability of this conclusion necessitates further verification in future investigations with larger sample sizes. In summary, the combined use of SII and blood coagulation indexes exhibits substantial predictive value for TIC in patients with severe trauma. This predictive value may be attributed to their comprehensive assessment of inflammatory response, immune status, and blood coagulation function. In light of the study’s findings, healthcare practitioners in trauma care should integrate the systemic immune inflammation index (SII) with standard coagulation assessments (APTT, PT, FIB, D-Dimer) for early detection of traumatic coagulopathy in severe trauma patients. By closely monitoring these indicators, clinicians can obtain crucial references for accurately predicting the occurrence of TIC. Future research avenues should focus on validating the combined SII and coagulation index model in larger, multicenter cohorts to enhance its generalizability. Longitudinal studies tracking SII and coagulation dynamics may elucidate the temporal relationship between inflammation, immunity, and coagulation post-trauma. Lastly, incorporating emerging biomarkers and advanced analytical techniques, including machine learning algorithms, could refine the predictive accuracy and clinical utility of the model.

## Dodatak

### Conflict of interest statement

All the authors declare that they have no conflict of interest in this work.
